# Back to pluripotency: fully chemically induced reboot of human somatic cells

**DOI:** 10.1038/s41392-022-01109-5

**Published:** 2022-07-19

**Authors:** Lucas Lange, Miguel A. Esteban, Axel Schambach

**Affiliations:** 1 Institute of Experimental Hematology, Hannover Medical School, Hannover, 30625 Germany; 2 REBIRTH Research Center for Translational Regenerative Medicine, Hannover Medical School, Hannover, 30625 Germany; 3 Laboratory of Integrative Biology, Guangzhou Institutes of Biomedicine and Health, Chinese Academy of Sciences, Guangzhou, 510530 China; 4 Division of Hematology/Oncology, Boston Children’s Hospital, Harvard Medical School, Boston, MA 02115 USA

**Keywords:** Regenerative medicine, Stem-cell biotechnology

In a recent study published in *Nature*, Guan et al.^1^ showed an exciting strategy to reprogram human somatic cells into human chemically induced pluripotent stem cells (hCiPSCs). Thereby, they identified crucial steps and pathways that pose a barrier to chemically induced reprogramming of human cells.

Human induced pluripotent stem cells (iPSCs) are promising tools for regenerative medicine to restore or replace damaged tissue. Being similar to embryonic stem cells but individualized and devoid of ethical concerns, iPSCs open new avenues for disease modeling and autologous tailored cell therapy that could lead to novel curative treatment options for diseases currently untreatable. Since the first generation of iPSCs in 2006 by ectopic expression of the Yamanaka factors in murine somatic cells, many other groups have successfully followed the transcription factor (TF)-based strategy to induce pluripotency in human adult somatic cells. Despite the enormous potential of iPSCs for regenerative medicine, therapeutic application of iPSC-derived cell products remains challenging, and only a few iPSC-based clinical trials have been conducted.^2^ Thus, what are the highest barriers for the clinical translation of iPSCs?

One of the most pressing problems is the safety concern due to genomic instability and potential tumorigenic potential of iPSC-derived cell products, possibly attributed to the reprogramming process. The TF-mediated reprogramming can be categorized into either integrating or non-integrating vector systems, each with particular advantages and disadvantages. Integrating viral vectors (e.g. retroviral/lentiviral vectors) were initially used to reprogram somatic cells. Despite their relatively high reprogramming efficiency, safety concerns have been raised about possible integration-induced genotoxicity. These concerns led to robust, non-integrating viral (e.g. Sendai-viral vectors) and non-viral expression vectors (e.g. mRNA transfer) to avoid genetic alterations. However, these ‘safer’ strategies still rely on ectopic expression of potential oncogenes that might hamper the therapeutic use of iPSC-derived cells. Thus, methods to avoid ectopic expression of oncogenes, e.g. by activation of endogenous gene expression, are highly sought. In this direction, chemically induced reprogramming represents a significant advance toward a potentially safer, transgene-free generation of human iPSCs. Compared to most other viral and non-viral vectors, chemical compounds can be cost-effectively manufactured, are incredibly versatile, can be precisely controlled in dosage and timing, and can be easily combined with other factors to achieve synergistic effects. Earlier studies identified small molecules to substitute one or several of the Yamanaka factors or drastically enhance the efficiency of TF-mediated reprogramming. Likewise, murine iPSCs can be successfully reprogrammed using a combination of small-molecule compounds to generate murine CiPSCs.^3^ The generated CiPSCs demonstrated an embryonic stem cell-like gene expression profile, epigenetic status, and differentiation potential. The early stages of the chemical reprogramming were characterized by gene expression profiles similar to extra-embryonic endoderm (XEN). The same group further optimized the chemically induced reprogramming protocol for murine somatic cells and described the importance of the intermediate XEN-like stage. However, chemically induced reprogramming of human somatic cells remained elusive, indicating additional barriers that derail the reprogramming of human cells.

**Fig. 1:**
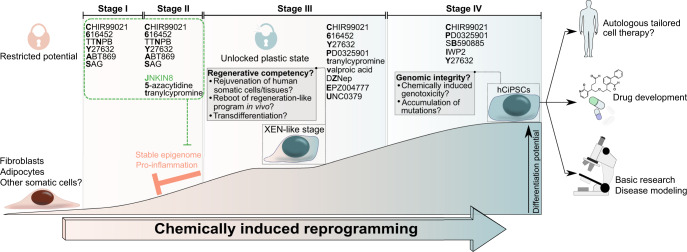
Schematic of chemically induced reprogramming of human somatic cells into human chemically induced pluripotent stem cells (hCiPSC). Starting from restricted, terminally differentiated human somatic cells, Guan et al. have established a fully defined, stepwise (stage I to stage IV) reprogramming protocol that relies entirely on small molecules. Disrupted somatic cell identity and epigenetic modulation induced dedifferentiation into a plastic XEN-like state with unlocked restricted potential. Especially the c-Jun N-terminal kinase inhibitor (JNKIN8) was indispensable to downregulate pro-inflammatory pathways that hinder dedifferentiation and generation of cell plasticity. The acquired cell plasticity of the XEN-like stage permitted further reprogramming to stable hCiPSCs. The hCiPSCs can be used for basic research, for example, to further investigate pathways involved in reprogramming, or to screen for cell fate-determining druggable targets that might lead to novel therapeutic options for patients. GMP-compliant, cost-effective reprogramming of human somatic cells into hCiPSCs potentially eases the translation of iPSCs for tailored autologous cell therapy

The JNK pathway is one of these barriers and it was also shown to impede the reprogramming of murine somatic cells.^4^ Guan et al. identified that inhibition of the JNK pathway and suppression of pro-inflammatory pathways (e.g. TNF/IL-1β) were indispensable for chemical reprogramming into hCiPSCs.^1^ In addition, they postulated that human cells possess a more stable epigenome that protects the cell identity from modification. Enhanced plasticity of human somatic cells by promoting DNA demethylation to increase chromatin accessibility, suppressing somatic cell identity, and upregulating genes needed for proliferation is crucial for the permissibility of chemical reprogramming. Based on the small molecules used for chemical reprogramming of murine somatic cells^3^ and screening for additional small molecules, the authors developed a fully defined and precisely staged chemically induced reprogramming protocol for human somatic cells (Fig. 1). The reprogramming was initiated with the addition of six small molecules to suppress somatic cell identity and activate a regeneration-like gene program (stage I), followed by addition of three more small molecules (stage II) to induce an epigenetic modulation towards a DNA hypomethylated and proliferative state. This further increased cell plasticity and enabled the formation of an intermediate cellular state similar to the XEN-like state in murine cells (stage III).

Interestingly, single-cell RNA-sequencing profiling of this intermediate plastic XEN-like state revealed upregulation of genes (e.g. *LIN28A* and *SALL4*) associated with developing human limb bud cells. Another report recently described the induction of sustained regenerative competency with upregulation of *Sall4* by brief exposure to small molecules in *Xenopus laevis*.^5^ Thus, could a chemical-induced XEN-like stage also reboot a regeneration-like program of specific tissues altered by an age-dependent decline of regenerative ability in humans? This intermediate plastic state was indispensable for the activation of the pluripotency gene network with additional small molecules (stage IV). The reprogramming efficiency of fetal and adult somatic cells into CiPSCs was up to 2.56%, and characterized by an embryonic stem cell-like transcriptome, epigenome and functionality in vitro and in vivo. Intriguingly, the reprogrammed CiPSCs transited through a naïve pluripotency state, but developed unstable genomic integrity under naïve culturing conditions. In primed culture conditions, CiPSCs resembled primed iPSCs and maintained genome integrity upon expansion for more than 20 passages.

Major obstacles to clinical translation of iPSC technology include genomic integrity issues and mutation accumulation during the reprogramming process and iPSC cultivation. Although the risk of genome instability of integrative reprogramming vectors might be higher, chemically induced genotoxicity is also a potential risk and should be carefully evaluated in chemically reprogrammed cells, especially after long-term cultivation. More specific assays will be needed to assess this possibility. The same applies to the DNA methylation state of the differentiated cells from CiPSCs. Whether global DNA demethylation induced by chemicals is carried over in the differentiated state and affects DNA stability remains to be tested. It will also be interesting to ascertain if the reprogramming of different somatic cell types will require different combinations of small molecules to unlock the restricted cell identity and induce CiPSCs. In this regard, some somatic cell types may be less amenable to chemically induced genotoxicity than others. In summary, this exciting method of fully chemically induced reprogrammed human somatic cells is a significant step towards generating defined and cost-effectively GMP-manufactured pluripotent cells. Moreover, the chemical reprogramming platform described by Guan et al. raises interesting questions concerning human tissue regeneration. Altogether, this work opens fascinating new possibilities for regenerative medicine.
